# Dietary Supplementation With Lily Bulb Core Extract Improves Feed Efficiency, Meat Quality, and Intestinal Health in Yellow‐Feathered Broilers

**DOI:** 10.1002/fsn3.72359

**Published:** 2026-09-19

**Authors:** Jiawei Tan, Jiahui Yin, Xiqing Fan, Linyu Zhang, Chenxia Xia, Shaodi Sun, Xiaoyu Mi, Yunhai Hu, Jianguo Zeng, Wei Xiang, Hongqi Xie

**Affiliations:** ^1^ Hunan Key Laboratory of Chinese Veterinary Medicine, College of Veterinary Medicine Hunan Agricultural University Changsha Hunan China; ^2^ Longhui County Agricultural Technology Extension Center Hunan Longhui Hunan China; ^3^ College of Horticulture Hunan Agricultural University Changsha Hunan China; ^4^ Chinese Medicinal Materials Breeding Innovation Center of Yuelushan Laboratory Changsha Hunan China

**Keywords:** carcass traits, functional feed additive, growth performance, gut microbiota, short‐chain fatty acid

## Abstract

Lily bulb core is an underused by‐product of edible lily processing. We hypothesized that dietary supplementation with lily bulb core extract (BHX) would improve growth performance and meat quality and would be accompanied by changes in intestinal morphology, cecal fermentation, and microbiota. A total of 240 one‐day‐old male yellow‐feathered broilers were assigned to four dietary treatments with six replicate cages of 10 birds each: a basal diet (CON) or the basal diet supplemented with 300, 600, or 900 mg/kg BHX for 53 days. All BHX treatments increased average daily gain over days 1–53, whereas 300 and 600 mg/kg reduced the feed conversion ratio compared with CON. Cooking loss in both breast and leg muscles was lower at 600 and 900 mg/kg than in CON. The duodenal villus‐height‐to‐crypt‐depth ratio was increased at 300 and 600 mg/kg. Cecal butyrate increased in all BHX groups and reached the highest concentration at 900 mg/kg. Overall alpha and beta diversity did not differ significantly among treatments, although selected bacterial genera differed among groups. BHX improved several production, meat‐quality, and intestinal traits, with 600 mg/kg producing the most consistent overall response under the present experimental conditions.

## Graphical abstract

Dietary lily bulb core extract improved feed efficiency, meat quality, and intestinal health in yellow‐feathered broilers. Supplementation at 600 mg/kg produced the most consistent overall benefits, enhancing average daily gain and the feed conversion ratio while reducing cooking loss in both breast and leg muscles. BHX treatment also improved intestinal villus morphology, increased cecal short‐chain fatty acid concentrations, and shifted microbiota composition toward beneficial genera including *Alistipes*, *Akkermansia*, and CHKC10027 under the present experimental conditions.

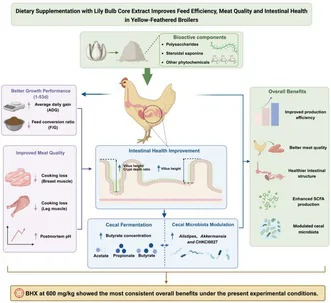

## 1 Introduction

Poultry meat is an important source of dietary protein worldwide, with projections indicating that it will account for more than 45% of global meat consumption by 2026 (Obianwuna et al. 2024). As demand for safe broiler meat grows, the ban on antibiotic growth promoters (AGPs) in China since 2020 has created an urgent need for effective alternatives (J. Wang et al. 2024). In southern China, where high temperatures and humidity characterize the subtropical climate, yellow‐feathered broilers represent a major local breed, and the transition to AGP‐free production requires locally adapted solutions (Gadde et al. 2017). The poultry industry concurrently faces increasing pressure to adopt sustainable practices that minimize environmental impact while maintaining production efficiency (Kleyn and Ciacciariello 2025).

Currently, numerous phytogenic additives (e.g., *Allium sativum* , *Origanum vulgare* , and *Agaricus bisporus*) have demonstrated growth‐promoting effects in broilers, yet these commonly used herbs require dedicated cultivation land, creating competition with human food resources and raising sustainability concerns (Aziz‐Aliabadi et al. 2024; Franciosini et al. 2016). Moreover, inconsistent bioactive composition due to geographical variations poses challenges for standardized industrial application (Obianwuna et al. 2024). In contrast, lily bulb core—a by‐product generated during the processing of edible lily bulbs—represents an underutilized agricultural residue that retains concentrated bioactive compounds given its central location within the bulb structure. This waste‐to‐value approach distinguishes BHX from conventional phytogenic supplements, offering environmental waste reduction and feed cost savings without additional land use (Mirabella et al. 2014; Roy et al. 2023).

Lily (*Lilium* spp.) is rich in bioactive compounds including polysaccharides and steroidal saponins, which have demonstrated various pharmacological activities (Hu et al. 2022; National Pharmacopoeia Commission 2020). The lily bulb core—the small inner scales typically discarded during processing—retains these concentrated bioactive constituents, yet its potential as a functional feed ingredient remains unexplored.

Therefore, we hypothesized that dietary supplementation with lily bulb core extract (BHX) would improve growth performance and meat quality and would be accompanied by changes in intestinal morphology, cecal short‐chain fatty acid (SCFA) production, and microbiota composition. Accordingly, the present study evaluated three BHX supplementation levels (300, 600, and 900 mg/kg) in yellow‐feathered broilers and determined their effects on growth performance, carcass traits, meat quality, duodenal morphology, cecal SCFAs, and cecal microbiota. Associations among microbial taxa, SCFAs, and phenotypic traits were further explored to generate hypotheses regarding the potential relationships underlying the observed responses.

## 2 Materials and Methods

### 2.1 Preparation of Lily Bulb Core Extract

The lily core was provided by Baoqing Longya Lily Development Co. Ltd. (Longhui County, Hunan Province, China), and the raw material was identified as Longya lily (*Lilium brownii* var. *viridulum* Baker). The aqueous extraction procedure was adapted from traditional Chinese medicine processing standards (D. Gao et al. 2022; Ministry of Agriculture and Rural Affairs of the People's Republic of China 2020). Briefly, dried lily core samples were ground to pass through a 60‐mesh sieve (0.250 mm). The extraction yield was calculated as the ratio of dry extract weight to raw material weight. Extraction was performed by heat‐reflux decoction with a material‐to‐liquid ratio of 1:20 (w/v). The lily core powder was soaked in ultrapure water for 2 h at room temperature (25°C ± 2°C), then subjected to a double‐cycle decoction (100°C, 1 h each cycle, with magnetic stirring at 300 rpm). After each decoction, the mixture was filtered through four layers of gauze (200 mesh, 0.074 mm) followed by centrifugation at 8000× *g* for 10 min at 4°C to remove insoluble residues. The combined supernatants were concentrated under reduced pressure at 60°C using a rotary evaporator (RE‐52AA, Yarong, China) until the volume was reduced to approximately 10% of the original, then vacuum‐dried at 60°C for 48 h (DZF‐6020, Yiheng, China) to obtain the lily core aqueous extract (BHX, brownish powder). The extraction yield was 18.5% ± 1.2% (w/w, *n* = 3 batches). The extract was stored at −20°C in airtight containers until use.

### 2.2 Analysis of Extract Components

The contents of total polysaccharides, total saponins, total polyphenols, and total flavonoids were determined using established spectrophotometric methods. Total polysaccharides were quantified by the phenol‐sulfuric acid colorimetric method (W. Li et al. 2020) with slight modifications. Briefly, 1 mL of appropriately diluted sample solution was mixed with 1 mL of 6% phenol solution and 5 mL of concentrated sulfuric acid. The mixture was vortexed for 30 s and incubated at room temperature (25°C ± 2°C) for 20 min. Absorbance was measured at 490 nm using a UV‐2600 spectrophotometer (Shimadzu, Kyoto, Japan). A calibration curve was prepared using glucose standards (0.02–0.2 mg/mL, *R* ^2^ > 0.99), and results were expressed as glucose equivalents. Total saponins were measured by the vanillin‐perchloric acid colorimetric method with slight modifications (Y. Chen et al. 2007). Briefly, 0.5 mL of sample solution was mixed with 0.2 mL of 5% vanillin‐glacial acetic acid solution and 1.2 mL of perchloric acid (70%). The mixture was incubated in a water bath at 70°C for 15 min, cooled to room temperature in an ice bath, and diluted with 5 mL of glacial acetic acid. Absorbance was measured at 560 nm. A calibration curve was prepared using oleanolic acid standards (0.01–0.1 mg/mL, *R* ^2^ > 0.99), and results were expressed as oleanolic acid equivalents. Total polyphenols were determined by the Folin–Ciocalteu method (Mohammed et al. 2022). Briefly, 0.2 mL of sample solution was mixed with 1.5 mL of Folin–Ciocalteu reagent (diluted 1:10 with distilled water) and 1.2 mL of 7.5% (w/v) sodium carbonate solution. The mixture was vortexed and incubated at room temperature in the dark for 2 h. Absorbance was measured at 765 nm. A calibration curve was prepared using gallic acid standards (0.01–0.5 mg/mL, *R* ^2^ > 0.99), and results were expressed as gallic acid equivalents (mg GAE/g extract). Total flavonoids were quantified by the aluminum chloride colorimetric method (Qian et al. 2025). Briefly, 1 mL of sample solution was mixed with 0.3 mL of 5% (w/v) sodium nitrite solution and incubated at room temperature for 6 min; then 0.3 mL of 10% (w/v) aluminum chloride solution was added and incubated at room temperature for 6 min; finally, 2 mL of 1 M sodium hydroxide solution was added and the mixture was diluted to 10 mL with distilled water. The mixture was vortexed and incubated at room temperature for 15 min. Absorbance was measured at 510 nm. A calibration curve was prepared using rutin standards (0.005–0.1 mg/mL, *R* ^2^ > 0.99), and results were expressed as rutin equivalents (mg RE/g extract).

### 2.3 Animals, Experimental Design, and Diets

Animal care and experimental procedures complied with the Chinese Animal Welfare Guidelines and were approved by the Ethics Committee of Hunan Agricultural University, China (No. 2025226). A total of 240 healthy one‐day‐old male yellow‐feathered broilers were obtained from Hunan Jitai Agriculture and Animal Husbandry Co. Ltd. (Changsha, China) and randomly assigned to four treatments, with six replicate cages of 10 birds per treatment. The design was selected with reference to comparable poultry nutrition studies and practical replicate requirements (C. Liu, Huang, et al. 2024; Long et al. 2020). Birds received a basal diet (CON) or the basal diet supplemented with 300, 600, or 900 mg/kg BHX (BHX‐L, BHX‐M, and BHX‐H, respectively). The doses were based on an unpublished pilot study (*n* = 60) and ranges reported for plant polysaccharides and lily‐derived extracts (C. Li, Zhu, et al. 2024; C. Liu, Huang, et al. 2024). The 600 mg/kg treatment represented the intermediate dose, whereas 300 and 900 mg/kg were included to characterize the response across a wider supplementation range. Diets were formulated according to the Chinese Chicken Feeding Standard (NY/T 33–2004) and were provided as starter diets from days 1 to 28 and grower diets from days 29 to 53 (Table 1). BHX was first mixed with 5 kg of carrier premix for 15 min and was then incorporated into the basal diet by sequential dilution and final mixing for 15 min. Mixing uniformity was not analytically verified. The replicate cage was the experimental unit for growth performance (*n* = 6 per treatment). Birds were housed under controlled conditions (33°C–35°C days 1–7; 22°C–25°C, days 8–28; 18°C–22°C, days 29–53), with a relative humidity of 60%–70% initially and 50%–60% thereafter, and continuous lighting. Routine vaccinations were performed by the chick supplier and during the feeding trial according to the commercial vaccination program and local veterinary recommendations. Total mortality during the experiment was below 1.5%.

**TABLE 1:** Dietary ratios and nutrient levels of experimental broiler feeds (air‐dried basis).

Item	Content	
	1~28 days	29~53 days
Corn, %	52.30	60.20
Soybean meal, %	39.40	30.00
Soybean oil, %	4.00	6.00
Dicalcium phosphate, %	2.00	1.64
Limestone, %	1.03	1.10
L‐Lysine, %	0.12	0.02
DL‐Methionine, %	0.25	0.14
Premix^a^, %	0.90	0.90
Total, %	100.00	100.00
Metabolizable energy (MJ/kg)^b^	12.39	13.21
Crude protein, %	21.52	18.02
Lysine, %	1.30	0.96
Methionine, %	0.57	0.42
Methionine cysteine, %	0.91	0.71
Calcium, %	0.91	0.84
Total phosphorus, %	0.72	0.62

^a^ The premix provides per kilogram of feed. VA, 11550 IU; VD3, 4200 IU; VE, 28 IU; VK, 2.8 mg; VB1, 2.1 mg; VB2, 8.75 mg; VB6, 3.5 mg; VB12, 0.021 mg; D‐Biotin, 0.105 mg; VB9, 1.4 mg; Niacin, 42 mg; D‐Pantothenic acid, 11.2 mg; Cu (as copper sulfate) 8 mg; Fe (as ferrous sulfate) 100 mg; Mn (as manganese sulfate) 120 mg; I (as potassium iodide) 0.7 mg; Se (as sodium selenite) 0.3 mg; Zn (as zinc sulfate) 80 mg.

^b^ Metabolic energy is a calculated value, while the rest are measured values.

### 2.4 Measurement of Growth Performance

The experiment lasted for 53 days. On days 1, 28, and 53, broiler chickens were fasted for 12 h (water was available ad libitum) before being weighed. Daily feed intake was recorded. Average daily gain (ADG, g/day), average daily feed intake (ADFI, g/day), and the feed conversion ratio (F/G, g feed/g gain) were calculated according to the following formulas:

ADG (g/day) = (Final body weight, g − Initial body weight, g)/Number of days.

ADFI (g/day) = Total feed intake per replicate, g/Number of days/Number of birds per replicate.

F/G (g/g) = Total feed intake per replicate, g/Total weight gain per replicate, g.

### 2.5 Sample Collection

All samples were collected on day 53 of the experiment, when yellow‐feathered broilers reached typical market age. After a 12‐h fasting (with free access to water), two birds per replicate were randomly selected. For measurements obtained from the two sampled birds within each replicate cage, values were averaged at the cage level before statistical analysis. Birds were humanely euthanized by cervical dislocation. Cecal contents (approximately 5 g) were aseptically collected into sterile cryovials, snap‐frozen in liquid nitrogen, and stored at −80°C for subsequent SCFA analysis and 16S rRNA gene sequencing. Simultaneously, mid‐segments of the duodenum (approximately 3 cm each) were flushed with physiological saline, then fixed in 4% paraformaldehyde for histomorphological analysis. It should be noted that 16S rRNA gene sequencing was performed by Majorbio Bio‐Pharm Technology Co. Ltd. (Shanghai, China), concurrently with Zhang (Zhang et al. 2025), but with distinct experimental groups.

### 2.6 Slaughter Performance and Organ Indices

Slaughter performance and organ indices were determined according to the Chinese agricultural industry standard NY/T 823‐2020. Terminology for Poultry Production Performance Rankings and Statistical Measurement Methods (Ministry of Agriculture and Rural Affairs of the People's Republic of China 2020). After a 12‐h feed withdrawal with free access to water, two birds per replicate were weighed (live weight, LW) and humanely euthanized by cervical dislocation. Following blood collection, birds were scalded at 60°C for 90 s, defeathered, and manually eviscerated.

Carcass measurements were recorded as follows:

Carcass yield (%) = (Carcass weight/Live weight) × 100, where carcass weight = live weight minus blood, feathers, and internal organs (excluding lungs and kidneys).

Half‐eviscerated yield (%) = (Half‐eviscerated weight/Live weight) × 100, where half‐eviscerated weight = live weight minus blood, feathers, trachea, esophagus, gastrointestinal tract, spleen, pancreas, gallbladder, and reproductive organs.

Eviscerated yield (%) = (Eviscerated weight/Live weight) × 100, where eviscerated weight = half‐eviscerated weight minus head, metatarsals, and lungs/kidneys.

Breast muscle yield (%) = (Breast muscle weight/Eviscerated weight) × 100.

Leg muscle yield (%) = (Thigh and drumstick muscle weight/Eviscerated weight) × 100.

Abdominal fat yield (%) = (Abdominal fat pad weight/Eviscerated weight) × 100.

Organ indices were calculated as follows:

$$\text{Organ index}\;\left(\%\right)=\left[\text{Organ weight}\;\left(g\right)/\text{Live weight}\;\left(g\right)\right]\times 100.$$

All weights were recorded using an electronic balance (precision ±0.01 g; Model JA5003, Shanghai Precision Scientific Instrument Co. Ltd., China). Organ weights included the heart, liver, spleen, lungs, kidneys, thymus, and bursa of Fabricius.

### 2.7 Meat Quality Measurements

Breast and leg muscle samples were collected from two birds per replicate on day 53. Muscle pH was measured at 45 min, 24 h, and 48 h postmortem using a calibrated portable pH meter (Testo 205, Testo, Germany). Meat color parameters (L*, a*, and b*) were measured at the same time points using a colorimeter (CR‐400, Konica Minolta, Japan). Drip loss and cooking loss were determined using the gravimetric method and expressed as percentages. Shear force was measured in newtons (N) using a digital muscle tenderness meter (C‐LM3; Tenovo International Co. Ltd., Beijing, China) by shearing the cooked samples perpendicular to the muscle fiber direction.

### 2.8 Short‐Chain Fatty Acid Capacity Measurement

Cecal contents were collected from two birds per replicate cage and analyzed for SCFA concentrations by gas chromatography with flame ionization detection (GC‐FID) following established protocols (Abbiss et al. 2023; Konanov et al. 2022) with modifications. Briefly, 1.0 g of cecal chyme was homogenized in 5.0 mL of ultrapure water and acidified with 25% metaphosphoric acid (9:1, v/v) containing 2‐ethylbutyric acid (2.0 mM) as the internal standard. The mixture was vortexed for 30 s, incubated at 4°C for 12 h, and centrifuged at 10,000× *g* for 10 min at 4°C. The supernatant was collected, and the precipitate was extracted again with 4.0 mL of ultrapure water. The supernatants were combined, diluted to a final volume of 10 mL, and filtered through a 0.22 μm membrane filter (Millipore, Billerica, MA, USA) prior to injection.

SCFA analysis was performed using a gas chromatograph (GC‐2010 Plus, Shimadzu Corporation, Kyoto, Japan) equipped with a flame ionization detector (FID) and a DB‐FFAP capillary column (30 m × 0.25 mm i.d., 0.25 μm film thickness; Agilent, Santa Clara, CA, USA). The operating conditions were as follows: injector temperature 250°C, detector temperature 280°C, and column temperature programmed from 80°C (held for 1 min) to 200°C at a rate of 10°C/min. Helium was used as the carrier gas at a flow rate of 1.0 mL/min. The split ratio was 10:1, and the injection volume was 1.0 μL. Individual SCFAs (acetic, propionic, butyric, isobutyric, valeric, and isovaleric acids) were identified by comparison with authentic standards (Shimadzu Corporation, Kyoto, Japan) based on retention times and quantified using the internal standard method. Calibration curves (*R* ^2^ > 0.99) were constructed for each SCFA, and results were expressed as mg/g of cecal content.

### 2.9 Intestinal Indicator Measurement

#### 2.9.1 Histological Assessment of the Duodenum

Mid‐segments of the duodenum (approximately 3 cm) were collected from two birds per replicate cage, rinsed with physiological saline, fixed in 4% paraformaldehyde solution, and stained with hematoxylin and eosin (H&E). Villus height and crypt depth were measured, and the villus‐height‐to‐crypt depth ratio was calculated.

#### 2.9.2 Cecal Microbiota Analysis

Cecal contents were collected from two birds per replicate cage on day 53. Cecal contents from the two sampled birds within each cage were pooled to generate one composite sample per cage, resulting in six samples per treatment (*n* = 6). The pooled samples were immediately snap‐frozen in liquid nitrogen and stored at −80°C until 16S rRNA gene sequencing. The collected cecal content samples were sent to Majorbio Bio‐Pharm Technology Co. Ltd. for microbial diversity sequencing. It should be noted that the 16S rRNA gene sequencing for this study was performed concurrently with a separate investigation examining *Polygonatum odoratum* effects (Zhang et al. 2025), but the two studies used distinct experimental treatments and biological samples.

### 2.10 Data Processing and Statistical Analysis

Data were analyzed by one‐way analysis of variance (ANOVA) using IBM SPSS 26.0 (IBM Corp., Armonk, NY, USA). The replicate cage was considered the experimental unit for all statistical analyses. For measurements obtained from two birds within each cage, the two values were averaged to obtain one cage‐level value before statistical analysis (*n* = 6 cages per treatment). Normality and homogeneity of variances were confirmed by the Shapiro–Wilk and Levene tests, respectively (*p* > 0.05). *F*‐values, degrees of freedom (df), exact *p*‐values, and partial eta‐squared (*ηp* ^2^) are reported for all ANOVA analyses. Duncan's multiple range test was applied for post hoc pairwise comparisons when ANOVA showed a significant main effect (*p* < 0.05). Significance was set at *p* < 0.05, and high significance at *p* < 0.01. For microbiota data, alpha diversity was compared using the Kruskal–Wallis test, and differences in microbial community composition among treatments were assessed by analysis of similarities (ANOSIM) with 999 permutations based on Bray–Curtis dissimilarities. Principal coordinates analysis (PCoA) was performed for visualization. Spearman's rank correlation analysis was used to assess associations among genus‐level microbial abundances, SCFA concentrations, and phenotypic traits. Mantel tests were used to evaluate associations between microbial community dissimilarity and SCFA or phenotypic variables, with significance assessed by 999 permutations.

Microbiome Analysis. DNA was extracted using E.Z.N.A. Soil DNA Kit (Omega). The 16S V3‐V4 region was amplified with primers 338F (5′‐ACTCCTACGGGAGGCAGCAG‐3′)/806R (5′‐GGACTACHVGGGTWTCTAAT‐3′), purified, and sequenced on Illumina NextSeq 2000 (2 × 300 bp) by Majorbio (Shanghai, China). Libraries were prepared using the NEXTFLEX Rapid DNA‐Seq Kit. Raw sequencing reads were quality‐filtered using fastp, merged using FLASH, clustered into OTUs at 97% sequence similarity using UPARSE, and screened for chimeric sequences using UCHIME. Taxonomic classification was performed using the Ribosomal Database Project (RDP) Classifier against the SILVA database (v138). Subsequent analyses were conducted on the Majorbio Cloud platform (Lundberg et al. 2013).

## 3 Results

### 3.1 Composition Analysis Results

As shown in Table 2, the polysaccharide content in the lily bulb core extract was 33.22%, the total saponin content was 3.61%, the total polyphenol content was 0.59%, and the total flavonoid content was 0.27%.

**TABLE 2:** Main chemical composition of lily bulb core extract.

Components	%
Polysaccharides	33.22
Total saponins	3.61
Total polyphenols	0.59
Total flavonoids	0.27

### 3.2 Effects on Growth Performance

BHX supplementation affected several growth‐performance outcomes (*p* < 0.05, Table 3). At day 28, body weight, ADG, and ADFI were higher in all BHX groups than in CON (*p* < 0.01), whereas the F/G was lower only in BHX‐L (*p* < 0.05). At day 53, body weight and ADG over days 1–53 were higher in all BHX groups than in CON, and the F/G was lower in BHX‐L and BHX‐M (*p* < 0.05). No differences in ADG, ADFI, or F/G were detected among treatments during days 29–53 (*p* > 0.05).

**TABLE 3:** Effects of lily bulb core extract (BHX) on the growth performance of yellow‐feathered broilers.

Item	CON	BHX‐L	BHX‐M	BHX‐H	*F*	SEM	*p*	*ηp* ^2^
**Body weight, g**								
1 day	58.83	59.58	59.03	58.31	0.210	0.541	0.888	0.031
28 days	769.93^b^	897.19^a^	869.28^a^	877.97^a^	7.043	14.355	0.002	0.514
53 days	2074.83^b^	2207.00^a^	2194.67^a^	2186.33^a^	3.772	18.377	0.027	0.361
**1–28 days**								
Average daily weight gain, g/day	25.40^b^	29.95^a^	28.98^a^	29.28^a^	7.638	0.505	0.001	0.534
Average daily feed intake, g/day	50.90^b^	56.12^a^	54.54^a^	55.63^a^	5.618	0.630	0.006	0.457
F/G	2.01^a^	1.88^b^	1.89^ab^	1.90^ab^	3.635	0.018	0.031	0.353
**29–53 days**								
Average daily weight gain, g/day	52.19	52.39	53.01	52.33	0.166	0.421	0.918	0.024
Average daily feed intake, g/day	123.09	123.09	125.33	125.34	0.310	3.098	0.818	0.044
F/G	2.43	2.35	2.37	2.39	0.961	0.017	0.430	0.126
**1–53 days**								
Average daily weight gain, g/day	38.04^b^	40.52^a^	40.30^a^	40.15^a^	3.915	0.342	0.024	0.370
Average daily feed intake, g/day	86.78	88.25	88.44	89.06	0.567	0.623	0.643	0.078
F/G	2.28^a^	2.17^b^	2.20^b^	2.22^ab^	4.228	0.013	0.018	0.388

*Note:* Values are presented as means, with the standard error of the mean (SEM) shown in a separate column (*n* = 6 replicate cages per treatment). Degrees of freedom for all ANOVA analyses were (3, 20). Within a row, means with different lowercase superscript letters (a, b) differ significantly (*p* < 0.05), while means with the same or shared superscript letters (e.g., ab) do not differ significantly (*p* > 0.05). Data were analyzed by one‐way ANOVA, and Duncan's multiple range test was applied as post hoc comparison when the ANOVA showed a significant main effect (*p* < 0.05).

### 3.3 Effects on Slaughter Performance and Organ Index

Slaughter performance results (Table 4) showed significant effects of BHX supplementation on carcass yield and half‐eviscerated yield. The BHX‐M and BHX‐H groups had higher carcass yields than the CON group, whereas the BHX‐L group showed an intermediate value. A similar pattern was observed for half‐eviscerated yield. No significant differences were detected among treatments in eviscerated yield, breast muscle yield, leg muscle yield, abdominal fat yield, or organ indices (*p* > 0.05).

**TABLE 4:** Effect of lily bulb core extract (BHX) on slaughtering performance and organ index of yellow‐feathered broilers.

Item	CON	BHX‐L	BHX‐M	BHX‐H	*F*	SEM	*p*	*ηp* ^2^
Carcass yield, %	89.94^b^	90.63^ab^	91.32^a^	91.72^a^	3.530	0.240	0.034	0.346
Half‐eviscerated yield, %	81.97^b^	83.28^ab^	84.48^a^	84.64^a^	4.106	0.363	0.020	0.381
Eviscerated yield, %	67.83	68.49	69.58	69.29	0.827	0.431	0.494	0.11
Breast muscle yield, %	13.74	14.20	14.63	15.19	1.553	0.258	0.232	0.189
Leg muscle yield, %	20.75	19.69	19.81	22.11	1.757	0.445	0.188	0.209
Abdominal fat yield, %	2.97	3.05	3.37	2.44	0.804	0.212	0.506	0.108
**Organ Index**								
Heart, %	0.49	0.47	0.51	0.47	0.560	0.011	0.648	0.077
Liver, %	2.10	2.03	1.83	2.20	1.973	0.059	0.151	0.228
Spleen, %	0.19	0.17	0.19	0.17	0.238	0.012	0.869	0.034
Lung, %	0.47	0.50	0.52	0.50	0.262	0.020	0.852	0.038
Kidney, %	0.57	0.52	0.55	0.54	0.349	0.019	0.790	0.050
Thymus, %	0.49	0.57	0.61	0.50	0.371	0.041	0.775	0.304
Bursa of fabricius, %	0.21	0.22	0.20	0.23	2.719	0.014	0.072	0.009

*Note:* Values are presented as means, with the standard error of the mean (SEM) shown in a separate column (*n* = 6 replicate cages per treatment). Degrees of freedom for all ANOVA analyses were (3, 20). Within a row, means with different lowercase superscript letters (a, b) differ significantly (*p* < 0.05), while means with the same or shared superscript letters (e.g., ab) do not differ significantly (*p* > 0.05). Data were analyzed by one‐way ANOVA, and Duncan's multiple range test was applied as post hoc comparison when the ANOVA showed a significant main effect (*p* < 0.05).

### 3.4 Effects of Lily Bulb Core Extract on Meat Quality

As shown in Table 5, breast‐muscle cooking loss differed among treatments (*p* = 0.003), with lower values in the BHX‐M and BHX‐H groups than in CON, whereas BHX‐L showed an intermediate value. Breast‐muscle a* at 48 h (*p* = 0.001), b* at 48 h (*p* = 0.032), and pH at 48 h (*p* = 0.009) also differed among treatments. In leg muscle, b* at 24 h (*p* = 0.004), pH at 45 min (*p* = 0.003), drip loss (*p* = 0.018), and cooking loss (*p* < 0.001) were affected by BHX supplementation. The lowest leg‐muscle cooking loss was observed in the BHX‐H group. No significant differences were detected in the remaining meat color, pH, drip loss, or shear force measurements (*p* > 0.05).

**TABLE 5:** Effects of BHX supplementation on breast and leg muscle quality of yellow‐feathered broilers.

Item	CON	BHX‐L	BHX‐M	BHX‐H	*F*	SEM	*p*	*ηp* ^2^
**Breast muscle color**								
L*_45min_	59.14 ± 3.66	59.06 ± 3.35	57.90 ± 1.85	54.89 ± 2.31	2.891	0.592	0.053	0.237
a*_45min_	7.39 ± 1.44	6.79 ± 1.43	7.02 ± 0.77	6.35 ± 0.81	0.950	0.219	0.430	0.092
b*_45min_	10.23 ± 1.86	10.57 ± 1.47	10.98 ± 0.91	9.83 ± 1.87	0.577	0.278	0.635	0.058
L*_24h_	58.72 ± 3.57	59.73 ± 2.28	57.66 ± 2.62	59.50 ± 1.92	0.805	0.484	0.502	0.079
a*_24h_	9.99 ± 1.62	8.83 ± 0.96	9.19 ± 0.89	8.55 ± 1.14	2.246	0.229	0.105	0.194
b*_24h_	14.25 ± 1.44	14.27 ± 1.27	15.26 ± 1.52	14.68 ± 1.53	0.783	0.248	0.514	0.077
L*_48h_	58.40 ± 2.64	57.70 ± 2.26	56.53 ± 2.26	58.29 ± 1.73	0.924	0.407	0.442	0.09
a*_48h_	8.97 ± 1.06^a^	8.09 ± 0.77^a^	7.96 ± 0.40^ab^	7.03 ± 0.43^b^	8.804	0.204	0.001	0.435
b*_48h_	14.85 ± 1.67^ab^	15.72 ± 1.06^a^	14.90 ± 1.40^ab^	13.51 ± 1.10^b^	3.390	0.264	0.032	0.266
**pH**								
pH_45min_	6.05 ± 0.24	6.17 ± 0.37	6.28 ± 0.45	6.05 ± 0.39	0.604	0.062	0.618	0.061
pH_24h_	5.65 ± 0.84	5.71 ± 0.97	5.75 ± 0.22	5.84 ± 0.98	2.887	0.024	0.053	0.236
pH_48h_	5.75 ± 0.12^b^	5.86 ± 0.10^ab^	5.91 ± 0.06^a^	5.88 ± 0.06^a^	4.674	0.019	0.009	0.334
Drip loss, %	4.60 ± 1.07	4.16 ± 0.92	4.58 ± 1.04	4.15 ± 0.83	0.426	0.181	0.737	0.057
Cooking loss, %	22.93 ± 1.80^a^	20.06 ± 2.20^ab^	17.71 ± 1.49^b^	18.87 ± 2.68^b^	5.970	0.490	0.003	0.417
Shear force, *N*	21.20 ± 5.29	18.38 ± 3.58	19.44 ± 3.56	22.20 ± 5.46	0.762	0.877	0.526	0.084
**Leg muscle color**								
L*_45min_	63.03 ± 2.39	61.55 ± 2.54	60.32 ± 2.79	61.92 ± 3.67	1.253	0.497	0.309	0.118
a*_45min_	10.11 ± 1.65	10.76 ± 1.16	10.62 ± 1.64	10.70 ± 1.48	0.394	0.253	0.758	0.041
b*_45min_	9.66 ± 2.92	10.42 ± 1.21	11.22 ± 1.23	10.99 ± 1.52	0.987	0.349	0.413	0.096
L*_24h_	58.99 ± 3.51	58.30 ± 3.05	58.14 ± 6.14	60.90 ± 2.86	0.687	0.677	0.568	0.069
a*_24h_	13.47 ± 2.53	13.53 ± 1.60	13.78 ± 1.02	12.64 ± 1.19	0.453	0.313	0.717	0.046
b*_24h_	12.50 ± 1.38^b^	12.22 ± 1.68^b^	15.16 ± 1.47^a^	13.07 ± 1.34^b^	5.497	0.316	0.004	0.371
L*_48h_	56.60 ± 3.37	56.43 ± 2.56	57.92 ± 0.30	58.58 ± 2.99	0.108	0.490	0.108	0.192
a*_48h_	13.75 ± 1.99	13.81 ± 1.92	12.60 ± 0.97	11.93 ± 0.85	2.247	0.311	0.105	0.194
b*_48h_	12.36 ± 1.68	12.78 ± 1.99	11.57 ± 1.95	10.19 ± 1.77	2.725	0.354	0.063	0.226
**pH**								
pH_45min_	5.93 ± 0.20^b^	6.25 ± 0.18^a^	6.21 ± 0.24^a^	6.31 ± 0.15^a^	5.962	0.040	0.003	0.390
pH_24h_	6.26 ± 0.20	6.25 ± 0.16	6.10 ± 0.30	6.29 ± 0.14	1.034	0.036	0.393	0.100
pH_48h_	5.75 ± 0.12	5.86 ± 0.10	5.91 ± 0.06	5.88 ± 0.06	1.624	0.036	0.206	0.148
Drip loss, %	3.94 ± 2.14^a^	2.06 ± 1.23^b^	3.02 ± 0.95^ab^	2.45 ± 1.15^ab^	3.722	0.232	0.018	0.096
Cooking loss, %	23.05 ± 3.80^a^	21.42 ± 2.62^ab^	18.33 ± 2.87^bc^	15.25 ± 2.67^c^	8.955	0.724	< 0.001	0.508
Shear force, *N*	17.96 ± 2.54	18.90 ± 6.09	17.54 ± 2.88	20.21 ± 4.19	0.620	0.690	0.608	0.067

*Note:* Values are means ± standard deviation (SD) (*n* = 6 replicate cages per treatment). Degrees of freedom for all ANOVA analyses were (3, 20). Within a row, means with different lowercase superscript letters (a, b) differ significantly (*p* < 0.05), while means with the same or shared superscript letters (e.g., ab) do not differ significantly (*p* > 0.05). Data were analyzed by one‐way ANOVA, and Duncan's multiple range test was applied as a post hoc comparison when the ANOVA showed a significant main effect (*p* < 0.05).

### 3.5 Effects on Cecal Short‐Chain Fatty Acids

BHX supplementation affected cecal butyrate concentration (*p* < 0.001, Table 6). Butyrate increased from 1.02 mg/g in CON to 1.29 mg/g in both BHX‐L and BHX‐M and to 1.48 mg/g in BHX‐H. Isobutyrate was lower in BHX‐L and BHX‐M than in CON, whereas BHX‐H did not differ from CON. Isovalerate was higher only in BHX‐H. Acetate, propionate, and valerate did not differ among treatments (*p* > 0.05).

**TABLE 6:** Effects of BHX supplementation on cecal short‐chain fatty acid concentrations in yellow‐feathered broilers.

Item	CON	BHX‐L	BHX‐M	BHX‐H	*F*	SEM	*p*	*ηp* ^2^
Acetic acid, mg/g	7.93 ± 1.24	6.77 ± 0.18	7.29 ± 1.33	7.53 ± 1.72	0.446	0.334	0.727	0.143
Propionic acid, mg/g	1.51 ± 0.24	1.18 ± 0.87	1.38 ± 0.05	1.52 ± 0.31	1.853	0.064	0.216	0.410
Isobutyric acid, mg/g	0.27 ± 0.01^a^	0.20 ± 0.10^c^	0.24 ± 0.01^b^	0.26 ± 0.01^a^	37.630	0.008	< 0.001	0.934
Butyric acid, mg/g	1.02 ± 0.01^c^	1.29 ± 0.03^b^	1.29 ± 0.07^b^	1.48 ± 0.03^a^	65.842	0.050	< 0.001	0.961
Valeric acid, mg/g	0.46 ± 0.11	0.51 ± 0.03	0.58 ± 0.10	0.62 ± 0.10	1.982	0.029	0.195	0.426
Isovaleric acid, mg/g	0.29 ± 0.01^b^	0.29 ± 0.02^b^	0.28 ± 0.01^b^	0.33 ± 0.02^a^	7.682	0.007	0.010	0.742

*Note:* Values are presented as means ± standard deviation (SD) (*n* = 3). Degrees of freedom for all ANOVA analyses were (3, 8). Means within a row with different lowercase superscripts differ significantly (*p* < 0.05), while means with the same superscript do not differ significantly (*p* > 0.05). Data were analyzed by one‐way ANOVA, and Duncan's multiple range test was used for multiple comparisons when the main effect was significant (*p* < 0.05).

### 3.6 Effects on Intestinal Morphology

**FIGURE 1:**
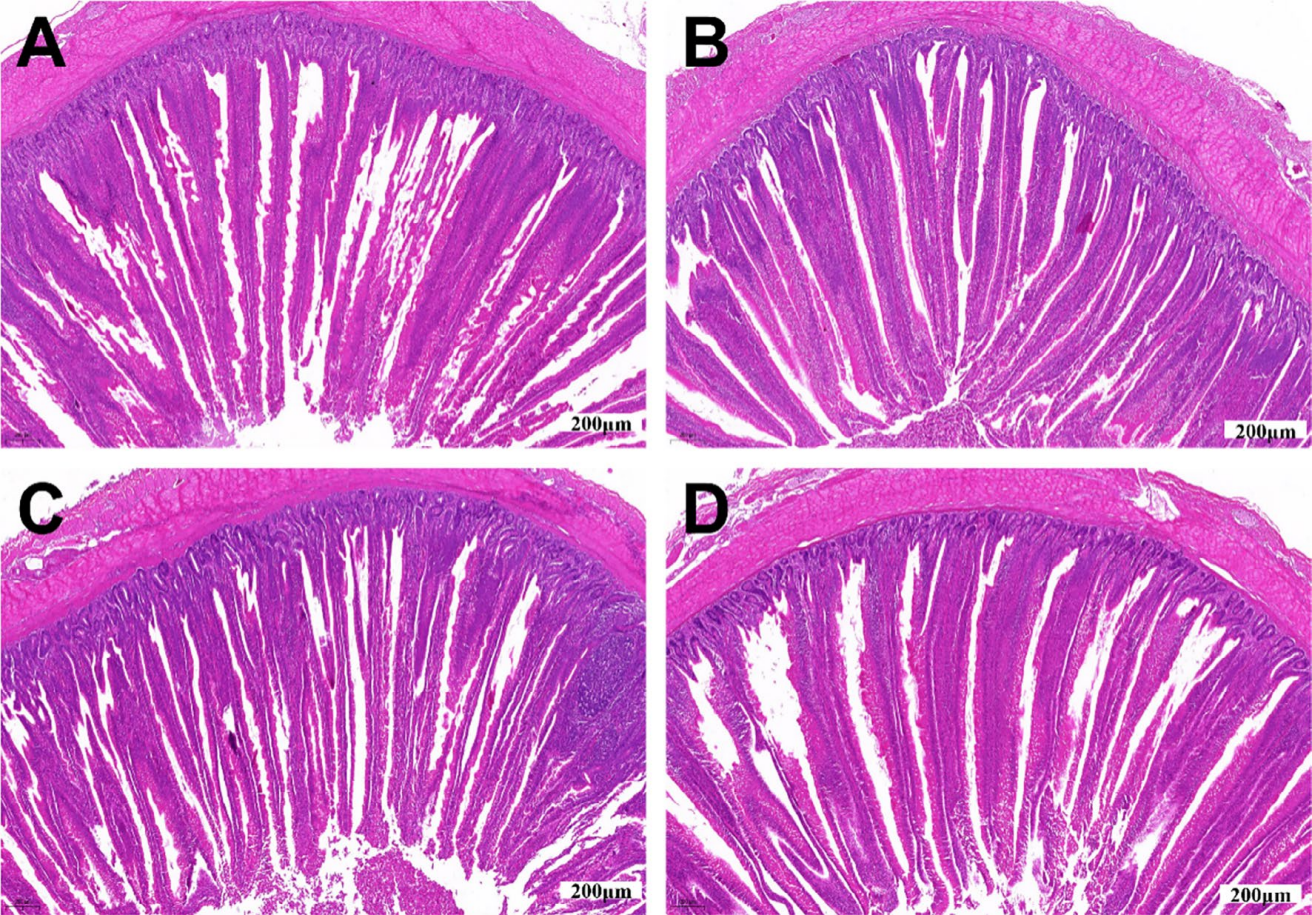
Morphological structure of the duodenum in yellow‐feathered broilers. Duodenal morphology of yellow‐feathered broilers in A–D: CON, BHX‐L, BHX‐M, and BHX‐H groups (HE staining, 5×).

Representative images in Figure 1 show generally comparable villus architecture among the treatment groups, with no obvious differences in villus length. The effects of BHX on duodenal morphology are shown in Table 7. There were no significant differences in duodenal villus length and crypt depth among the groups (*p* > 0.05). However, the villus‐height‐to‐crypt‐depth ratio differed significantly among treatments (*p* = 0.008), with higher values observed in the BHX‐L and BHX‐M groups than in CON.

**TABLE 7:** Effects of BHX supplementation on duodenal morphology in yellow‐feathered broilers.

Item	CON	BHX‐L	BHX‐M	BHX‐H	*F*	SEM	*p*	*ηp* ^2^
Villus height, μm	1528.69	1465.89	1452.43	1558.27	2.864	17.329	0.072	0.364
Crypt depth, μm	138.78	124.97	133.60	131.96	2.367	2.010	0.112	0.321
Villi/crypt, ratio	10.23^c^	11.28^ab^	11.78^a^	10.59^bc^	5.828	0.189	0.008	0.538

*Note:* Values are presented as means, with the standard error of the mean (SEM) shown in a separate column (*n* = 6 replicate cages per treatment). Degrees of freedom for all ANOVA analyses were (3, 20). Means within a row with different lowercase superscripts differ significantly (*p* < 0.05), while means with the same superscript do not differ significantly (*p* > 0.05). Data were analyzed by one‐way ANOVA, and Duncan's multiple range test was used for multiple comparisons when the main effect was significant (*p* < 0.05).

### 3.7 Cecal Microbiota Diversity

**FIGURE 2:**
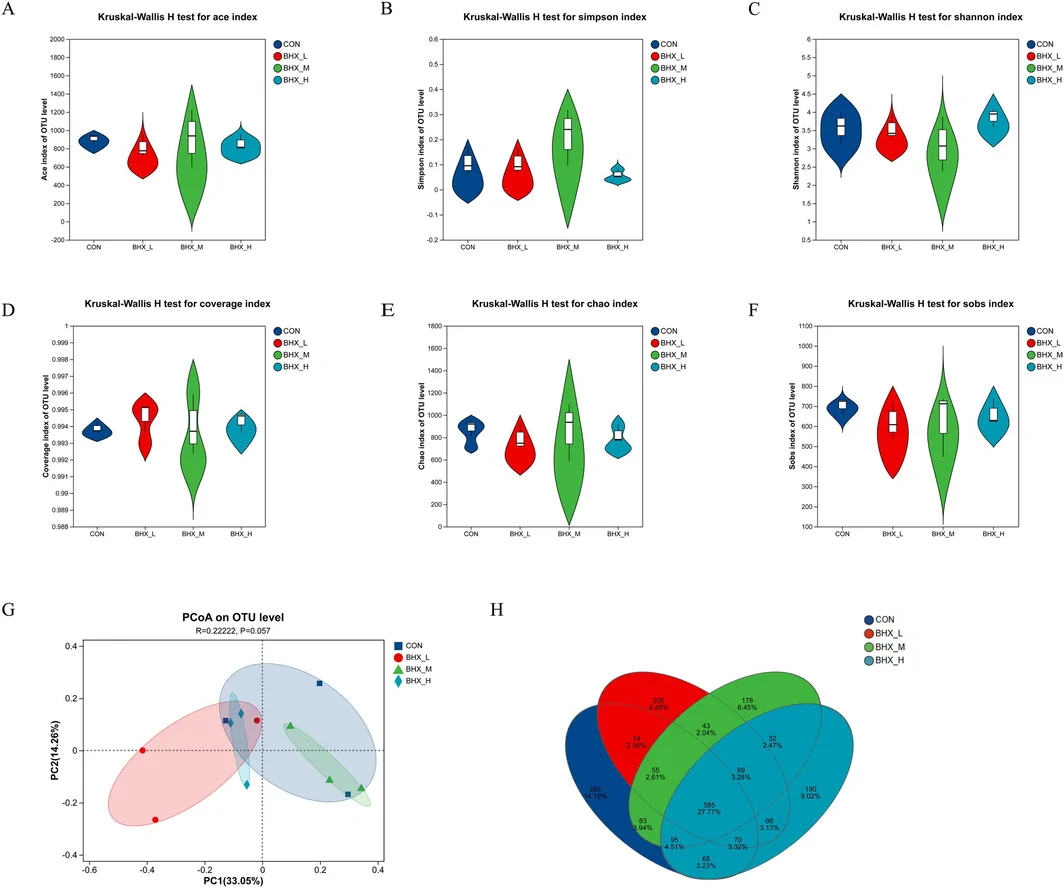
Effect of BHX supplementation on gut microbiota diversity. (A–F) Chao, Sobs, Shannon, Simpson, Coverage, and Shannoneven indexes of microflora on operational taxonomic unit (OTU) level; (G) Principal co‐ordinates analysis (PCoA) for microflora on OTU level. The *p*‐value was obtained by ANOSIM for testing the significance of separation between sample groups. (H) Venn diagram of intestinal microflora in each group.

As noted in the Methods section, sequencing for this study was performed concurrently with Zhang et al. (2025), but the two studies analyzed completely independent experimental groups. Alpha‐diversity metrics, including the Ace, Simpson, Shannon, Coverage, Chao, and Sobs indices, were evaluated. As shown in Figure 2A–F, no significant differences were detected among treatments for any alpha‐diversity index (*p* > 0.05). A PCoA based on Bray–Curtis distances showed separation among groups, but β‐diversity differences were not significant (*R* = 0.222, *p* = 0.057; Figure 2G). The Venn diagram showed 585 OTUs shared by all four treatments. The numbers of treatment‐specific OTUs were 289, 207, 178, and 190 for CON, BHX‐L, BHX‐M, and BHX‐H, respectively (Figure 2H).

### 3.8 Relative Abundance Analysis of Cecal Microbiota

**FIGURE 3:**
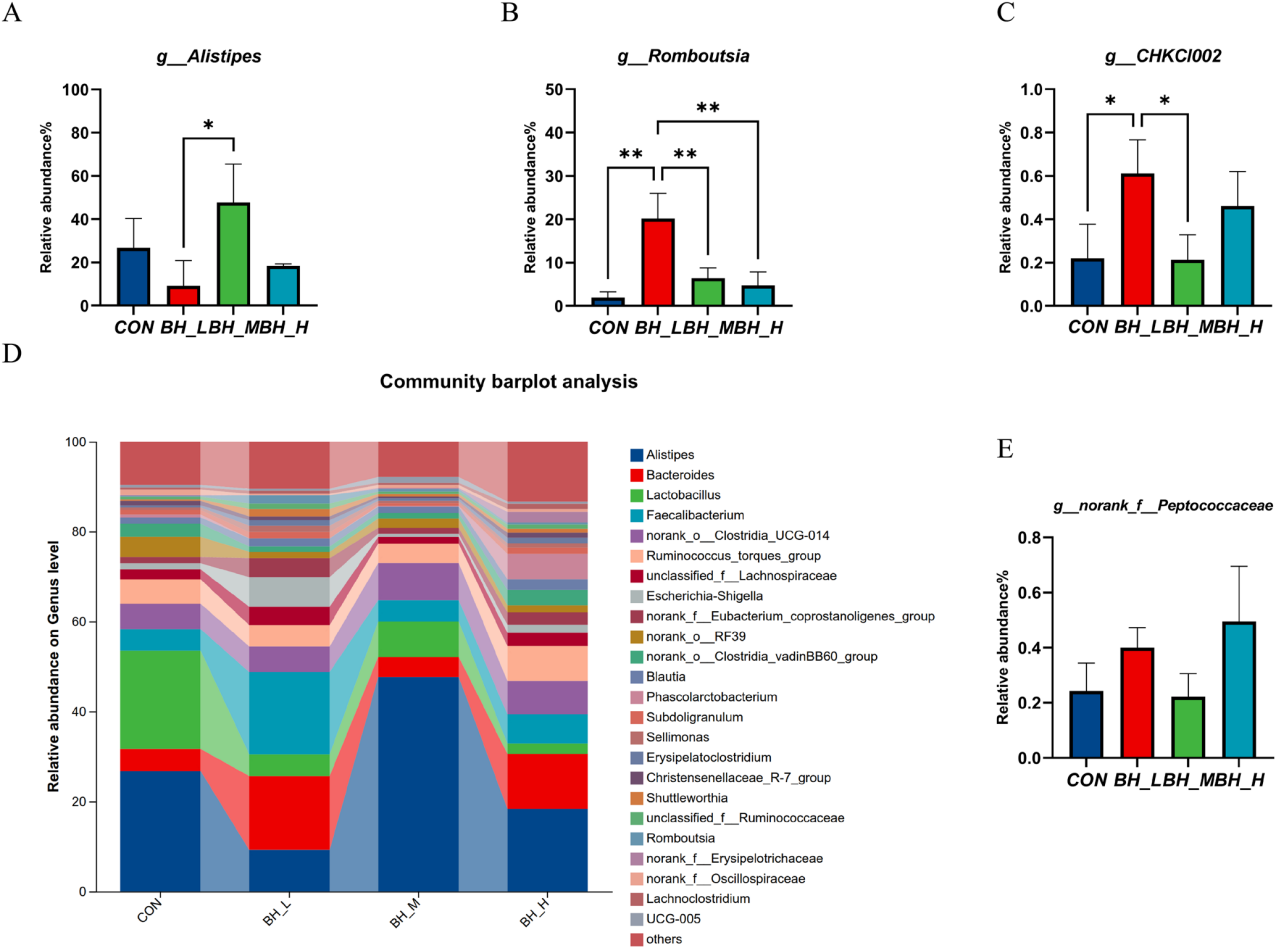
Effects of lily bulb extract on the intestinal microbiota of yellow‐feathered broilers at the genus level. (A) Comparison of relative abundance of *Alistipes* across treatment groups; (B) Comparison of relative abundance of *Romboutsia* across treatment groups; (C) Comparison of relative abundance of *CHKCI002* across treatment groups; (D) Community barplot analysis of intestinal microbiota at the genus level, depicting dynamic shifts in species composition across treatment groups; (E) Comparison of relative abundance of *norank_f_Peptococcaceae* across treatment groups. *N* = 3 for each group. *p < 0.05; **p < 0.01.

To investigate the effects of BHX on the gut microbiota, relative abundance was analyzed at the genus level. The relative abundance of *Alistipes* was higher in BHX‐M than in BHX‐L (*p* < 0.05), whereas no other pairwise differences were indicated (*p* > 0.05, Figure 3A). The genus *Romboutsia* (Figure 3B) was significantly enriched in the BHX‐L group compared with the CON, BHX‐M, and BHX‐H groups (*p* < 0.01). CHKCI002 (Figure 3C) was most abundant in the BHX‐L group, which was significantly higher than that in the CON and BHX‐M groups (*p* < 0.05). The relative abundance of *norank_f_Peptococcaceae* was numerically highest in BHX‐H, but the difference was not statistically significant (*p* > 0.05, Figure 3E).

Community barplot analysis (Figure 3D) revealed distinct patterns across groups. The CON group was dominated by *Alistipes*, *Lachnospiraceae*, and *Bacteroides*. The BHX‐L group showed marked increases in *Romboutsia* and CHKCI002, whereas *Alistipes* was reduced. The BHX‐M group was characterized by the enrichment of *Alistipes* and a reduction of *Romboutsia*. The BHX‐H group exhibited elevated levels and a partial recovery of *Alistipes* compared with the BHX‐L group.

### 3.9 Associations of Cecal Microbiota, Short‐Chain Fatty Acids, and Phenotypic Traits

**FIGURE 4:**
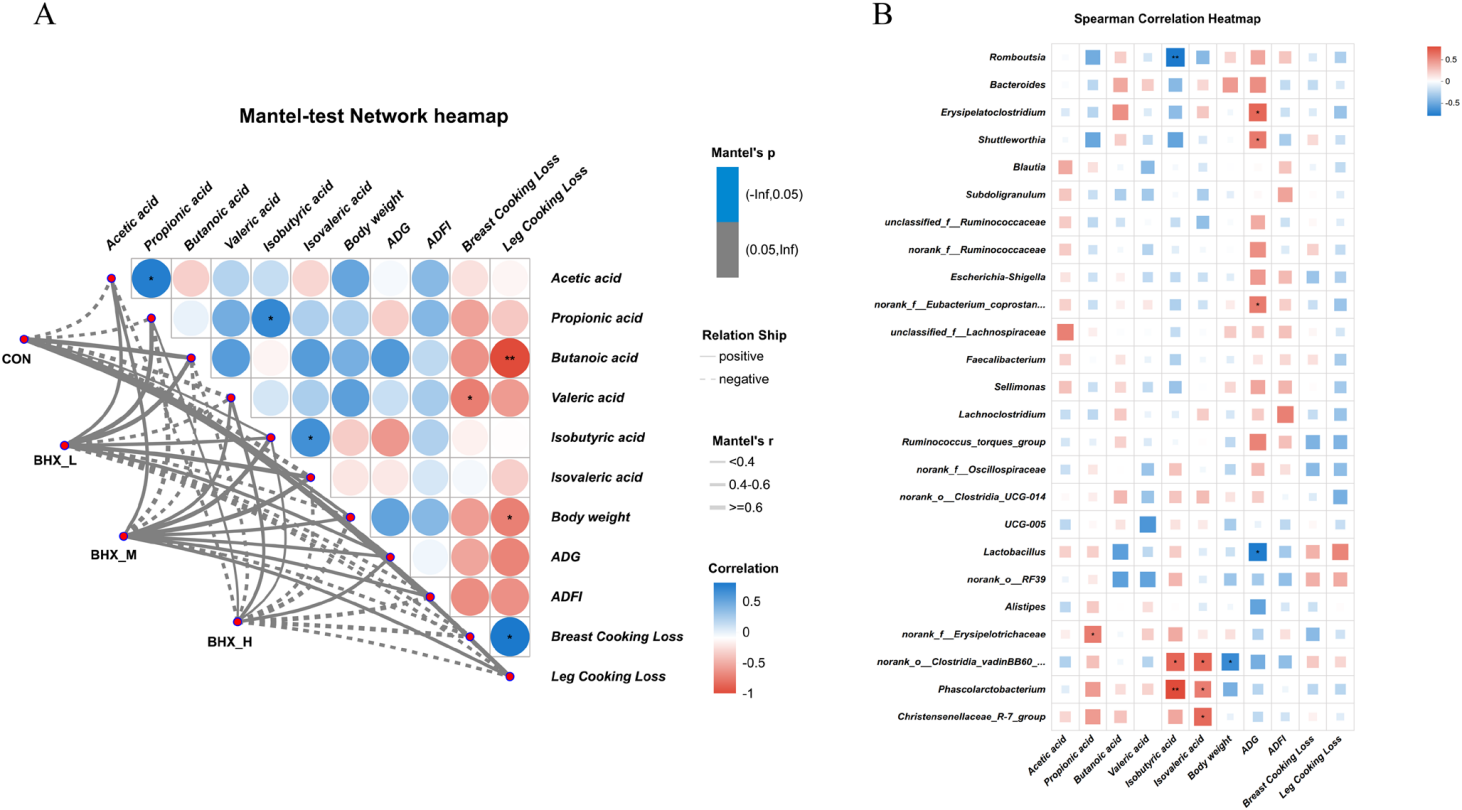
Correlation analysis of intestinal microbiota, metabolites, and growth performance indices. (A) Mantel‐test network heatmap, illustrating the correlations between intestinal microbiota, metabolites, growth performance, and meat quality traits; (B) Spearman correlation heatmap, showing the strength and direction of associations between key microbial genera, metabolites, and phenotypic indices.

Mantel tests indicated significant associations between overall cecal microbiota composition and several SCFAs and phenotypic traits (Figure 4A). Associations with butyrate, isobutyrate, body weight, and breast‐muscle cooking loss showed Mantel's *r* > 0.4 (*p* < 0.05). Spearman correlation analysis further identified genus‐specific associations (Figure 4B). *Butyricicoccus* and *Romboutsia* were negatively correlated with isobutyrate, whereas *Akkermansia* and *norank_f_Peptococcaceae* were positively correlated with butyrate. Additional significant correlations were observed between *norank_f__Erysipelotrichaceae* and propionate and between *Tyzzerella* and valerate. Both *Phascolarctobacterium* and *norank_o__Clostridia_vadinBB60_group* were significantly correlated with isobutyrate and isovalerate, whereas *Christensenellaceae_R‐7_group* and *unclassified_c__Clostridia* were correlated with isobutyrate. *Butyricimonas* was also significantly correlated with isovalerate (*p* < 0.05).

## 4 Discussion

### 4.1 Effects on Growth Performance and Dose Response

Dietary BHX increased ADG over days 1–53, while 300 and 600 mg/kg reduced the F/G without increasing overall ADFI. This pattern indicates that BHX improved the conversion of consumed feed into body gain rather than stimulating feed intake. Similar growth‐promoting effects have been reported for plant polysaccharides in broilers and other animal models (Long et al. 2020; Ren et al. 2017). Water extract of *Chuanminshen violaceum* stems and leaves likewise increased ADG and reduced the F/G in broilers (H. Liu, Zhang, et al. 2024). Comparable responses have also been observed with *Achyranthes japonica* extracts under different dietary conditions (Khan et al. 2024; Muniyappan et al. 2022; Sun et al. 2020). The convergence of these botanically distinct preparations on feed efficiency points to the intestine as a shared physiological target.

BHX contained 33.22% crude polysaccharides and 3.61% total saponins. A likely explanation is that part of the polysaccharide fraction escaped upper‐intestinal digestion and entered the cecum as a fermentable substrate. The accompanying increase in cecal butyrate and redistribution of selected bacterial genera support this interpretation. The response was non‐linear: 300 and 600 mg/kg produced the most consistent improvements, whereas 900 mg/kg increased butyrate without providing additional growth benefits. This divergence suggests that the growth response reached a physiological ceiling once intestinal fermentation and nutrient utilization were sufficiently improved.

### 4.2 Effects on Slaughter Performance and Organ Indices

BHX‐M and BHX‐H increased carcass yield and half‐eviscerated yield, while breast and leg muscle yields remained unchanged. BHX therefore improved the conversion of live weight into carcass mass without inducing selective muscle hypertrophy. Nutritional interventions can produce different responses in growth, carcass allocation, and clinical traits (Jespersen et al. 2021), which may explain why the carcass response did not precisely parallel the muscle‐yield results.

Plant polysaccharides have been linked to PI3K/Akt/mTOR‐dependent growth and protein synthesis in other poultry models (S. Gao et al. 2025; Long et al. 2020). Muscle redox status can also influence energy metabolism and tissue development (Z. Chen et al. 2021). The increases in ADG, carcass yield, and half‐eviscerated yield were not accompanied by significant changes in breast or leg muscle yields. Organ indices were not significantly affected by BHX supplementation during the 53‐day feeding period. Together with previous evaluations of immune‐organ development and feed‐additive safety (Gadde et al. 2017; Qui 2022), these findings support the short‐term tolerability of BHX at the tested doses.

### 4.3 Impact on Meat Quality

BHX reduced cooking loss in both breast and leg muscles, altered selected postmortem pH measurements, and reduced leg‐muscle drip loss at 300 mg/kg. These coordinated changes indicate improved muscle water‐holding capacity. Similar improvements in meat‐quality traits have been reported for other phytogenic additives (Dang et al. 2022; Shanmugam et al. 2022; Yu et al. 2021). Postmortem pH regulates the charge and spatial organization of myofibrillar proteins, thereby determining their ability to retain water during storage and cooking (Puolanne and Halonen 2010). BHX probably modified postmortem acidification and stabilized water binding within the myofibrillar structure.

The increase in cecal butyrate provides a plausible metabolic link between intestinal fermentation and muscle quality. Oxidative stress disrupts redox balance and glycolytic metabolism in broiler breast muscle (Z. Chen et al. 2021), whereas tributyrin can improve muscle oxidative status through Nrf2‐related responses (C. Chen et al. 2024). Nrf2‐ and sirtuin‐dependent redox regulation also provides a broader framework for understanding the relationship between cellular metabolism and tissue quality (Corsello et al. 2018). BHX may therefore influence postmortem muscle metabolism through fermentation‐derived signals, with butyrate contributing to the observed improvement in water retention.

The color response was less uniform. Breast‐muscle a* at 48 h decreased with increasing BHX dose, whereas b* showed a non‐linear pattern and was higher in BHX‐M than in CON. Meat color is jointly determined by L*, a*, and b*, as well as by myoglobin chemistry and oxidative status (J. Li et al. 2019; Lynch and Faustman 2000). BHX therefore exerted a more consistent effect on muscle water‐holding capacity than on color stability.

### 4.4 Effects on Intestinal Morphology and Cecal SCFAs

BHX did not alter villus height or crypt depth individually, but 300 and 600 mg/kg increased their ratio. This change indicates a more favorable balance between the absorptive and proliferative compartments of the duodenal mucosa. The villus‐height‐to‐crypt depth ratio is widely used to evaluate intestinal functional architecture (Z. Li, Li, et al. 2024; Matos et al. 2025). Similar morphological responses have accompanied improved growth performance in broilers receiving phytogenic extracts or fermented diets (H. Liu, Zhang, et al. 2024; Ma et al. 2022). The improved mucosal architecture probably contributed to the higher ADG and lower F/G observed in the BHX‐L and BHX‐M groups.

SCFAs are generated through microbial fermentation of dietary substrates that escape host digestion (McCrory et al. 2024). BHX increased cecal butyrate at all doses, with the highest concentration at 900 mg/kg. Butyrate provides energy to intestinal epithelial cells and regulates intestinal homeostasis and immune activity (Anshory et al. 2023). Dietary interventions that improve intestinal morphology and inflammatory status can also enhance broiler performance (Zhou et al. 2025). These findings show that BHX supplementation was accompanied by increased cecal butyrate and changes in duodenal morphology; whether these responses are mechanistically linked requires further investigation.

The highest butyrate concentration did not coincide with the strongest growth response. This finding suggests a threshold effect: once sufficient butyrate was available to support intestinal function, further increases did not translate into additional body‐weight gain. Cecal butyrate therefore appears to be an important component of the BHX response, but not the sole determinant of growth performance.

### 4.5 Impact on Cecal Microbiota

The cecal microbiota supports nutrient fermentation, immune regulation, and metabolic communication with the host (Abd El‐Hack et al. 2022; Kamada et al. 2012). BHX did not significantly change overall alpha or beta diversity, indicating that it did not broadly reconstruct the microbial ecosystem. Instead, BHX selectively redistributed bacterial niches. *Alistipes* was more abundant in BHX‐M than in BHX‐L, whereas *Romboutsia* and CHKCI002 were enriched in BHX‐L. *Norank_f_Peptococcaceae* was numerically most abundant in BHX‐H.

Associations between the cecal microbiota and feed efficiency have been reported in chickens, including diet‐dependent relationships involving *Alistipes* (Bernard et al. 2024). Prebiotic‐induced changes in the cecal microbiome and metabolome have also been linked to meat characteristics (Yang et al. 2022). Other studies have connected microbial community composition with SCFA metabolism, intestinal homeostasis, and host performance (Gautam et al. 2024; Keerqin et al. 2021; Z. Li et al. 2021; Marcolla et al. 2023; L. Wang et al. 2016). These findings place the BHX‐responsive taxa within a broader network linking microbial fermentation to nutrient utilization and tissue phenotypes.

Butyrate increased across all BHX doses despite distinct genus‐level profiles. This pattern may be consistent with microbial functional redundancy, whereby different bacterial communities maintain a similar fermentation output. BHX therefore appears to regulate microbial function more consistently than overall taxonomic structure. Improvements in intestinal barrier function have also been reported for other phytogenic preparations, including essential‐oil blends (Sampath et al. 2025). Together, these observations suggest that BHX‐related changes in cecal fermentation, including increased butyrate production and shifts in selected microbial taxa, may be associated with improvements in intestinal and production outcomes.

### 4.6 Integrated Interpretation and Future Directions

Taken together, BHX supplementation was associated with improvements in growth performance, meat quality, duodenal morphology, cecal SCFAs, and selected microbial taxa. The dose response was non‐linear, with 600 mg/kg producing the most consistent overall benefits, whereas increasing the dose to 900 mg/kg did not confer additional growth advantages despite yielding the highest cecal butyrate concentration. This pattern suggests that the growth‐promoting effects of BHX may approach a plateau within the tested supplementation range. However, because nutrient digestibility, intestinal nutrient transport, and individual BHX constituents were not directly evaluated, the basis of this non‐linear response remains unclear. In addition, BHX was characterized mainly by UV–visible spectrophotometric assays at the class level; therefore, the observed biological effects cannot be attributed to any specific bioactive constituent. Moreover, the microbiota–SCFA–phenotype associations should be interpreted as hypothesis‐generating rather than causal. Future studies should further characterize the specific bioactive constituents of BHX and clarify their dose–response relationships and underlying mechanisms. Because only male broilers were evaluated, additional validation in female birds and under commercial production conditions is warranted.

## 5 Conclusion

Dietary lily bulb core extract (BHX) improved growth performance, meat quality, and intestinal morphology in yellow‐feathered broilers. Supplementation at 600 mg/kg produced the most consistent overall response, including increased average daily gain, reduced the F/G, increased carcass yield, and a higher duodenal villus‐height‐to‐crypt‐depth ratio. BHX supplementation also increased the cecal butyrate concentration and was associated with changes in the relative abundance of selected cecal bacterial genera, whereas overall microbial alpha and beta diversity remained unchanged. These findings suggest that 600 mg/kg BHX may be a promising supplementation level for yellow‐feathered broilers, pending further validation under commercial conditions.

## Author Contributions

**Jiawei Tan:** conceptualization, methodology, investigation, formal analysis, writing – original draft. **Jiahui Yin:** conceptualization, methodology, investigation, formal analysis, writing – original draft. **Xiqing Fan:** investigation. **Linyu Zhang:** investigation. **Chenxia Xia:** investigation. **Shaodi Sun:** investigation, data curation. **Xiaoyu Mi:** investigation. **Yunhai Hu:** resources. **Jianguo Zeng:** resources, experimental material preparation. **Wei Xiang:** supervision, writing – review and editing, technical guidance. **Hongqi Xie:** conceptualization, methodology, project administration, funding acquisition, supervision, writing – review and editing, resources.

## Funding

We would like to thank the Hunan Agriculture Research System (Grant No. HARS‐11) and Yuelu Mountain Laboratory Seed Industry Special Project Funding (YLS‐2026‐ZY01013) for funding this research.

## Ethics Statement

Animal care and procedures were carried out in accordance with the Chinese Animal Welfare Guidelines (State Council of the People's Republic of China 2017), and all experimental protocols were approved by the Ethics Committee of Hunan Agricultural University, China (No. 2025226). This study followed the ARRIVE guidelines 2.0 for reporting animal research (Percie du Sert et al. 2020).

## Conflicts of Interest

The authors declare no conflicts of interest.

## Data Availability

The data that support the findings of this study are available on request from the corresponding author. The data are not publicly available due to privacy or ethical restrictions.
